# Development and validation of TreatHSP-QoL: a patient-reported outcome measure for health-related quality of life in hereditary spastic paraplegia

**DOI:** 10.1186/s13023-023-03012-w

**Published:** 2024-01-02

**Authors:** Jekaterina Malina, Eva-Maria Huessler, Karl-Heinz Jöckel, Eva Boog-Whiteside, Nicole Jeschonneck, Bernadette Schröder, Rebecca Schüle, Tobias Kühl, Stephan Klebe

**Affiliations:** 1 Department of Neurology, University Hospital Essen, Essen, Germany; 2 Institute for Medical Informatics, Biometry and Epidemiology, University Hospital Essen, Essen, Germany; 3 Center for Clinical Trials, University Hospital Essen, Essen, Germany; 4 Division of Neurodegenerative Diseases, Department of Neurology, Heidelberg University Hospital and Faculty of Medicine, Heidelberg, Germany; 5 Center for Neurology and Hertie Institute for Clinical Brain Research, University of Tübingen, Tübingen, Germany; 6 German Center for Neurodegenerative Diseases (DZNE), Tübingen, Germany

**Keywords:** Health-related quality of life, Hereditary spastic paraplegia, Patient-centered outcome measure

## Abstract

**Background:**

Hereditary spastic paraplegia (HSP) is a rare neurodegenerative disease that lacks specific and validated patient-centered outcome measures (PCOMs). We aimed to develop and validate a health-related quality of life (HRQoL) questionnaire specific to HSP (“TreatHSP-QoL”) that could be used as a PCOM.

**Results:**

The pilot-items of the TreatHSP-QoL (45 five-level Likert scale items, with values per item between 0 and 4) were developed based on a qualitative data analysis of 54 semi-structured interviews, conducted in person with 36 HSP patients and 18 caregivers. It was then reduced and modified through the validation process to 25 items. The main validation was performed using the online questionnaire in 242 HSP patients and 56 caregivers. The exploratory factor analysis defined five subdomains. Cronbach’s alpha ranged from 0.57 to 0.85 for the subdomains and reached 0.85 for the total score. The test–retest Pearson correlation reached 0.86 (95% Confidence Interval (CI) [0.79, 0.91]). Pearson correlations with the EuroQol-5 Dimension (5 levels) (EQ-5D-5L) and Friedreich Ataxia Rating Scale-Activities of Daily Living (FARS-ADL) questionnaires varied strongly among the subdomains, with the total scores reaching 0.53 (95% CI [0.42, 0.61]) and -0.45 (95% CI [− 0.55, − 0.35]), respectively. The caregiver-patient response Pearson correlation ranged between 0.64 and 0.82 for subdomains and reached 0.65 (95% CI [0.38, 0.81]) for the total score.

**Conclusions:**

TreatHSP-QoL can be used in high-quality clinical trials and clinical practice as a disease-specific PCOM (i.e., HRQoL measure) and is also applicable as a proxy questionnaire. Score values between 0 and 100 can be reached, where higher value represents better HRQoL. The Pearson correlations to the EQ-5D-5L and FARS-ADL support the additional value and need of HSP-specific PCOM, while non-specific QoL-assessment and specific clinical self-assessment tools already exist. All in all, the results demonstrate good validity and reliability for this new patient-centered questionnaire for HSP.

**Supplementary Information:**

The online version contains supplementary material available at 10.1186/s13023-023-03012-w.

## Background

Hereditary spastic paraplegia (HSP) is a rare neurodegenerative disease, mainly characterized by lower limb weakness and spasticity, due to axonal degeneration of the corticospinal tract [1]. Prevalence varies widely, between 1.0 and 4.9 per 100,000 [2, 3]. Smaller studies report large regional differences with a prevalence of up to 19.9 per 100,000 [4]. All modes of inheritance (autosomal dominant, autosomal recessive, X-linked and, more rarely, mitochondrial inheritance) have been described [1]. With the recent advances in molecular genetic diagnostics, especially through next-generation, exome, and genome sequencing, more than 80 genetic causes for HSP (named SPG for Spastic Paraplegia Gene) have been identified in recent years [1, 5, 6]. The age at onset ranges from early childhood to over 70 years of age [1, 7]. Although dependent on the underlying genetic defect, this age is highly variable between family members with the same mutation for almost all HSP subtypes [6]. The clinical classification, based on historic description, distinguishes between pure and complex forms of HSP. Patients with pure HSP show isolated pyramidal signs, i.e., brisk reflexes, pyramidal signs, spasticity and motor deficits, which can be associated with sphincter disturbances and deep sensory loss [1, 5]. Complex forms consist of a multitude of clinical entities in which HSP is associated with variable combinations of other neurological or extra-neurological signs, such as cerebellar ataxia, dysarthria, mental retardation, peripheral neuropathy, optic atrophy, retinitis pigmentosa, hearing loss, or thin corpus callosum [1, 8, 9]. Due to genetic and clinical heterogeneity, the progression and prognosis in HSP patients is variable. The therapy of HSP is predominantly based on alleviation of symptoms (e.g., using spasmolytic therapy and physiotherapy) because no causal treatment exists.

New therapeutic approaches to rare genetic diseases have been developed in recent years. However – due to the character of rare diseases – many therapeutic studies have failed to prove clinical effectiveness or the statistical significance of the observed outcome differences [10]. A problem is the small number of cases of rare diseases, especially if they are genetically and clinically heterogeneous such as HSP. Another issue may be the lack of clinical trial readiness due to missing meaningful endpoints. An important question here is the impact of treatment-related effects on an individual daily function and whether existing tools are capable to measure this. The relevance of most available clinician-reported outcome measures and biomarkers to patients and their daily livings is not obvious as they do not capture patients’ own perspective and subjective well-being. Moreover, a very recent review of outcome measures and biomarkers for HSP showed variability and inconsistencies in use of outcome measures with a paucity of longitudinal data, highlighting the need for a standardized set of core outcome measures [11]. Regarding the clinical severity of HSP, the Spastic Paraplegia Rating Scale (SPRS) is a well-validated, clinician-reported scale that has been widely used since 2006 [12]. However, it does not reflect the perceived severity in patients. To develop effective treatments (symptomatic, disease-modifying, or causal), it is important to understand and quantify the disease impact that matters most to those who are affected. Accordingly, the International Rare Diseases Research Consortium concluded that developing patient-centered outcome measures (PCOMs) for rare diseases is a necessity [13]. So far PCOMs were only used infrequently to assess treatment outcomes in HSP-related studies [11]. PCOMs should be incorporated into high-standard treatment trials, but also used in clinical practice and in natural disease course studies, including disease registries. A suitable PCOM that respects the heterogeneity of rare diseases and clinical trial readiness is the health-related quality of life (HRQoL). This does not simply evaluate physical integrity, but also involves physical (individual physical perception), psychological (individual perception of the cognitive and affective state), and social (individual perception of the interpersonal) dimensions [14]. Specific validated HRQoL measures already exist for some common neurological movement disorders, e.g. the Parkinson Disease Questionnaire-39 for Parkinson's disease [15], which is used in well-structured studies as a primary or secondary outcome parameter [16, 17]. The lack of specific HRQoL-assessments is particularly seen in published studies on rare diseases in adults. For example, the studies on the efficacy of nusinersen in spinal muscular atrophy in adult patients did not use any HRQoL measure [18]. Generic, non-disease-specific HRQoL assessments are also being used in studies, e.g., for spinocerebellar ataxias [19, 20] or HSP [21, 22]. In HSP-related studies the most used HRQoL-assessment was the Short Form Health Survey-36 (SF-36) and its derivative, SF-12, followed by EuroQoL-5 Dimensions (EQ-5D) [11]. Siow et al. suggested the SF-36 as the most suitable PROM for HSP. However they noted that it may not be sensitive to smaller changes in HSP-specific symptoms, particularly when evaluating small changes in response to treatment in a slowly progressive condition [11]. A natural course 1-year-follow-up study in HSP comparing clinician- and patient-reported outcome measures, also suggested that generic PROMs (EQ-5D and Patient Health Questionnaire 9 (PHQ-9)) are not suitable to detect change in HSP and that a disease specific PROM is needed [23]. Thus, we aimed to overcome this unmet need for a HSP-specific HRQoL and develop a HRQoL questionnaire specific for HSP patients and a proxy questionnaire for their caregivers. The proxy questionnaire for caregivers can be particularly crucial for patients with complex forms of HSP, as they may not be able to complete questionnaires due to severe physical or intellectual disabilities.

## Results

### Item generation for the pilot questionnaire

Fifty-four interview transcripts (36 patient and 18 caregiver interviews), representing 37 different patients, were included in a qualitative data analysis (i.e., one caregiver was not related to an already-interviewed patient, unlike all other caregivers). Altogether, there were 21 (57%) male and 16 (43%) female patients whose age ranged from 18 to 74 years (Table 1). At this point, we noticed that the content was repetitive according to the different disease severities and enough data has been collected to draw necessary conclusions to perform data coding.

**Table 1:** Patient characteristics (patients with self-reported outcomes and those with caregiver-reported outcomes)

	Interviews		Pilot questionnaire	Main validation	
Reported by	Patients	Caregivers	Households	Patients	Caregivers
N	36	18	91	242	56
Age (mean [range])	53 [18, 74]	56 [18, 74]	52 [19, 79]	56 [21, 81]	50 [17, 85]
*Sex (N (%))*					
Female	15 (42)	8 (44)	40 (44)	129 (53)	28 (50)
Male	21 (58)	10 (56)	51 (56)	111 (46)	28 (50)
Diverse	0 (0)	0 (0)	0 (0)	2 (1)	0 (0)
SPRS (mean [range])	19 [4, 42]	22 [8, 42]	19 [4, 39]	–	–

*SPRS* Spastic Paraplegia Rating Scale

The genotypes were known for more than half of patients: six patients had SPG4 and six had SPG7; further genotypes were SPG5, SPG5A, SPG8, SPG10, SPG11, SPG31, and KIF1A. The rest were unknown (n = 15). The SPRS range for patients (out of a possible 0–52) was 4 to 42. The number of patients and their associated caregivers that were mildly, moderately, and severely affected can be found in Table 2.

**Table 2:** Disease severity of the interviewed patients and patients associated with the interviewed caregivers

Spastic paraplegia rating scale	Patients, n	Caregivers, n
0–15 (mild)	12	4
16–30 (moderate)	17	9
31–52 (severe)	7	5
Total	36	18

During the process of matrix coding of the transcripts, eight superordinate domains were identified: symptoms, mobility, assistance needs, leisure activities, social life, occupation, medical treatment, and attitude to the disease. A total of 41 pilot items were subsequently developed, mainly addressing the patient and caregiver issues through each domain (no. items): symptoms (seven), mobility (three), assistance needs (four), leisure (six), social life (six), occupation (four), medical treatment (six), attitude to the disease (five). Three out of 41 items were formulated differently in the patient and caregiver questionnaires, respecting the different wording in the interviews (e.g. "fear of wheelchair" as a patient item and "fear of advancing symptoms" as a corresponding caregiver item). Three additional items with free text fields were added to the symptoms domain, allowing participants to mention additional symptoms that were particularly important to them, if necessary. Furthermore, one superordinate item on general satisfaction with quality of life was added.

Altogether, the patient and caregiver pilot questionnaires contained 45 items each. All items were five-level Likert scale items, where either agreement/disagreement to a statement or level of disability or frequency of a state/an event could be rated. For some items, an additional answering option was provided in case the condition, or the statement did not apply to a particular participant (e.g., symptom items). At the end of the pilot questionnaires, three open questions were asked to collect feedback on the comprehensibility of the items (i.e., quality of wording), on missed topics (i.e., completeness of the questionnaire) and on the questionnaire in general.

### Pilot item validation

**Fig. 1:**
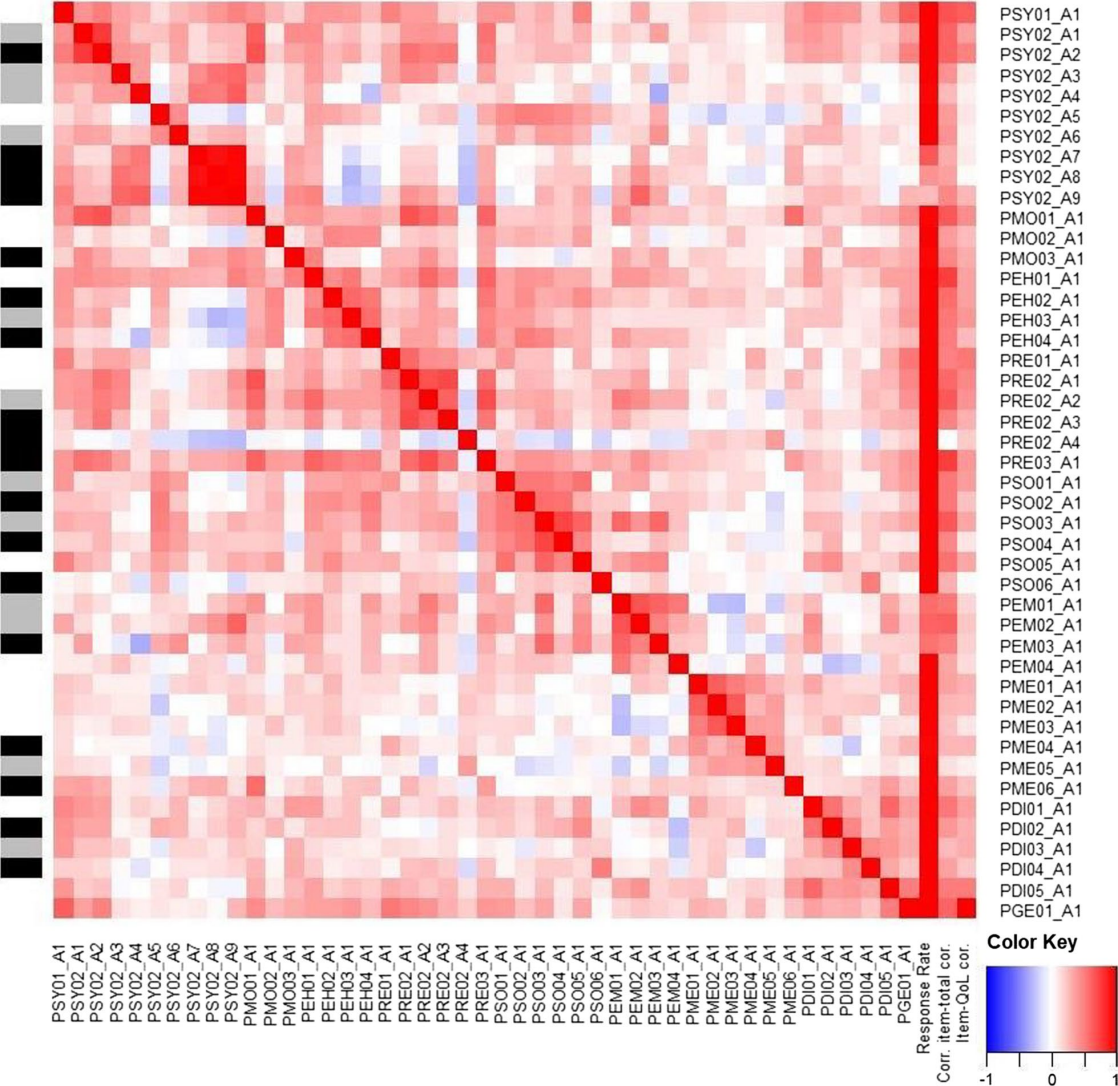
Heatmap of response rates and inter-item, corrected item-total and item-QoL correlations. The items are shown on the right and the bottom sidebar. Inter-item correlations are shown in the main body of the heatmap, with values between -1 (blue) and 1 (red). Response rates, corrected item-total and item-QoL correlations are shown on the right, with values between -1 (blue) and 1 (red) for correlations and values between 0 (white) and 1 (red) for response rates. The left side bar shows if the item was selected to be deleted (black), to be modified (gray), or to be unchanged (white)

For pilot item validation, the questionnaires were sent to 91 households. Within these 91 addressed households, there were 40 (44%) female and 51 (56%) male patients, with a mean age of 52 (range [16, 79]) years and a mean SPRS of 19 (range [4, 39]) (Table 1). The first validation was performed on 65 patients and 53 caregivers, giving a response rate of 71% and 58%, respectively. The items of the patient questionnaire were critically reviewed with respect to their validity and content. In summary, 18 items were then deleted, 12 were modified, and two new items were added. The variable selection and modification process is illustrated in the heatmap of the patient results with response rate, inter-item Pearson correlations, item-total Pearson correlation, and single-item Pearson correlation to the general quality of life question (Fig. 1). A detailed description of the process can be found in Additional file 1.

Finally, the modified patient questionnaire contained 29 items. Due to applied filter questions, patients could be distinguished between “working”, “non-working due to the disease”, and “non-working for another reason”. Depending on the occupational situation, patients would be referred to a different item, so that a maximum of 28 out of 29 items could be answered by each participant (for more details see Additional file 1).

The item difficulty for remaining items varied between 0.17 and 0.68 (standard deviations 0.85 to 1.47), implying that they were suitable for differentiation.

Validation of the caregiver items showed similar results to the patient items. Fifty-one of the caregiver pilot questionnaires could be linked to the corresponding patient questionnaires. The Pearson correlation between test scores of patients and their caregivers was 0.63 (95% Confidence Interval (CI) [0.43, 0.77]) when considering all items, and 0.66 (95% CI [0.46, 0.79]) when excluding deleted items. All of the 29 items, as well as the filter questions from the modified patient questionnaire, were transferred to a modified caregiver questionnaire.

### Main item validation

The modified questionnaire, implemented online in a patient and a caregiver version and distributed among HSP patient organization members, was started 315 times for patients and 91 times for caregivers. Uncompleted responses (i.e., finishing the questionnaire before reaching the last HRQoL item) were excluded from further analyses. A detailed overview of the number of replies for the patient and caregiver questionnaires, as well as for the retest questionnaires, can be found in the Additional file 2. Of the 67 patients who did not complete the questionnaire, 56 (84%) did not even start the HRQoL questionnaire and 64 (93%) only answered up to two items. Out of the 26 caregivers who did not complete the questionnaire, 21 (81%) did not even start the HRQoL questionnaire. Patient and caregiver responses where patients were < 16 years old were also excluded from further analyses because the item generation and modification process did not allow for children. In total, 242 patient and 56 caregiver responses remained for analyses. Thus, the exploratory factor analysis (EFA) could be conducted with more than 200 patient respondents and almost 9 respondents per item, counting 28 items, i.e. the maximum number of items per participant.

The age of the patients who self-administered the questionnaire was 56 (range [21, 81]) years on average. The age of the patients with questionnaires completed by caregivers was 50 (range [17, 85]) years on average. For the self-administered questionnaire, there were 129 (53%) female patients, 111 (46%) male patients, and 2 (1%) patients of diverse sex. For the caregiver-administered questionnaire, there were 28 (50%) female patients and 28 (50%) male patients. The disease duration of the patients was 21 (range [2, 71]) years on average for the patient self-administered questionnaires and 20 (range [2, 64]) years for the caregiver-administered questionnaires. Thirty-eight (67%) caregivers completed the questionnaire for their life partners, 11 (20%) for their children, 6 (11%) for other relatives, and one value (2%) was missing. Thirty-one (55%) caregiver responses could be matched to patient responses. An overview of patient characteristics during the main validation (along with patient characteristics during the interviews and pilot validation) is shown in Table 1.

The Kaiser–Meyer–Olkin criterion reached a value of 0.84, implying that data was suitable for exploratory factor analysis (EFA). In the EFA, there were seven factors with eigenvalues above one. Based on a scree plot, the first five factors were retained. By these five factors, more than 50% of the total variance in the data could be explained (i.e., 52.9%). The rotated factor loadings, which show the strength of the relationship between the item and the factor (i.e., subdomain), can be found in the Additional file 3. The five factors were identified as new subdomains according to the content of the included items: (I) general quality of life and attitude to the disease, (II) mobility and leisure time, (III) medical care, (IV) social life and occupation/work, and (V) associated symptoms. Only one item had factor loadings of less than 0.4 (item on need for help: PHE01_PHE02) and was therefore deleted for the final questionnaire. Three items had factor loadings of less than 0.5 and a difference between the two largest loadings of 0.15 or less. Two of them (items PSO01 and PMO02_PMO03) were deleted for the final questionnaire. The third item asked about pain (item PSY02_3), and had large factor loadings on the subdomains (II) mobility and leisure time and (V) associated symptoms. As pain was considered to have an important impact on patients’ lives, according both to the interviews and general medical knowledge, the item was retained.

For the confirmatory factor analysis (CFA), the items were assigned to the five factors as discovered in the EFA. When considering all items, the comparative fit index (CFI) was equal to 0.8029, the standardized root mean square residual (SRMR) to 0.0886, and the RMSEA to 0.0796. Deleting the item PSY02_3 in respect to its factor loadings (see above) yields in the CFI of 0.8210, a SRMR of 0.0821 and a root mean square error of approximation (RMSEA) of 0.0779, which showed no important difference to the first result and highlights the need to keep the item. Although the CFI value was smaller than general recommendations [24, 25], the SRMR and RMSEA showed a better model fit, with values close to 0.08 [25, 26].

**Fig. 2:**
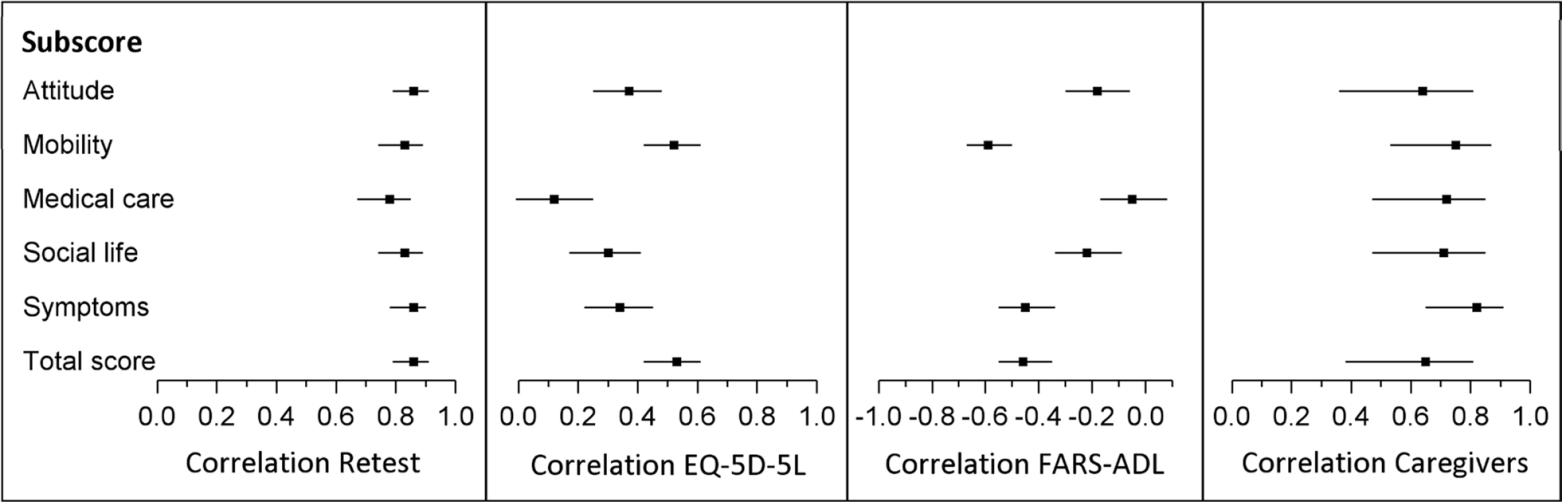
Forest plots with Pearson correlations to retest, EQ-5D-5L, FARS-ADL and the caregiver responses. The square points represent the Pearson correlations and the horizontal lines the 95% CIs. The results are shown for all subdomains and the total score. The names of the subdomains were reduced for visual reasons

Pearson correlations, Cronbach’s alpha, and retest reliability are represented in Table 3 and corresponding Fig. 2. Cronbach’s alpha was above 0.8 for the first three factors: (I) general quality of life and attitude to the disease, (II) mobility and leisure time, (III) medical care and for the total score. For the subdomain (V) associated symptoms, Cronbach’s alpha was relatively small (0.57). The Pearson correlations with the retest were above 0.8 for all subdomains, except for (III) medical care with a correlation of 0.78.

**Table 3:** Cronbach’s alpha (α), retest reliability, and Pearson correlations with EQ-5D-5L, FARS-ADL, and the caregiver questionnaire

	Items	Cronbach’s α	Retest [95% CI]	EQ-5D-5L [95% CI]	FARS-ADL [95% CI]	Caregivers [95% CI]
General QoL and attitude to disease	6	0.85	0.86 [0.79, 0.91]	0.37 [0.25, 0.48]	−0.18 [−0.30, −0.06]	0.64 [0.36, 0.81]
Mobility and leisure time	6	0.80	0.83 [0.74, 0.89]	0.52 [0.42, 0.61]	−0.59 [−0.67, −0.50]	0.75 [0.53, 0.87]
Medical care	4	0.80	0.78 [0.67, 0.85]	0.12 [−0.01, 0.25]	−0.05 [−0.17, 0.08]	0.72 [0.47, 0.85]
Social life and occupation/work	5	0.60	0.83 [0.74, 0.89]	0.30 [0.17, 0.41]	−0.22 [−0.34, −0.09]	0.71 [0.47, 0.85]
Associated symptoms	4	0.57	0.86 [0.78, 0.90]	0.34 [0.22, 0.45]	−0.45 [−0.55, −0.34]	0.82 [0.65, 0.91]
Total score	25	0.85	0.86 [0.79, 0.91]	0.53 [0.42, 0.61]	−0.46 [−0.55, −0.35]	0.65 [0.38, 0.81]

EQ-5D-5L, EuroQol-5 Dimension (5 levels); FARS-ADL, Friedreich Ataxia Rating Scale-Activities of Daily Living; QoL, quality of life

The Pearson correlations with the EQ-5D-5L were all positive and between 0.12 and 0.52 for subdomains and 0.53 for the total score. The highest correlations were reached for the subdomain (II) mobility and leisure time and the total score, while the lowest was with the subdomain (III) medical care.

The Pearson correlations with the FARS-ADL were negative and between − 0.05 and − 0.59 for subdomains and − 0.46 for the total score. The highest correlation of − 0.59 was reached with the subdomain (II) mobility and leisure time, while the lowest correlation was again with the subdomain (III) medical care.

The Pearson correlations between patient and caregiver responses were all above 0.6, i.e., between 0.64 and 0.82 for subdomains and 0.65 for the total score. All correlations between the corresponding patient and caregiver items were positive. There were three items in three different subdomains with correlations of < 0.3. When omitting these items, the subscore and total score correlations between patients and caregivers did not change for more than ± 0.03. Therefore, we decided to keep all items for the proxy questionnaire.

Thus, the final questionnaire contains 26 items. Taking into account the filter questions on occupational situation, a maximum of 25 items can be answered by each participant. The response distribution for the total score and the five subscores (regarding only remaining items for final questionnaires) can be found in Additional file 4.

The mean total score for the patient questionnaire was 56.6 (range [25.0, 92.0]) and for the caregiver questionnaire 51.3 (range [10.0, 80.0]). The mean patient score was 57.5 (range [26.0, 92.0]) for men and 56.0 (range [25.0, 92.0]) for women. For the caregiver questionnaire, the mean score was 48.7 (range [10.0, 80.0]) for men and 54.0 (range [29.0, 75.0]) for women. On average, the highest subscores were obtained for the associated symptoms subdomain, with a mean value of 79.1 for the patient questionnaire and of 75.1 for the caregiver questionnaire. The worst mean was obtained for the mobility and leisure time subdomain, with a mean subscore of 31.4 for the patient questionnaire and 29.4 for the caregiver questionnaire. There was a very low negative Pearson correlation (i.e., close to zero) of -0.13 (95% CI [-0.250, -0.002]) between the score and age for the patient questionnaire, and a very low positive correlation (0.11, 95% CI [-0.16, 0.36]) for the caregiver questionnaire. The Pearson correlation between the score and the disease duration was -0.06 (95% CI [-0.19, 0.07]) for the patient questionnaire and -0.01 (95% CI [-0.29, 0.28]) for the caregiver questionnaire. For every subdomain, very low (i.e., 0 or close to 0, representing a poor HRQoL) and very high subscores (i.e., 100 or close to 100, representing a good HRQoL) were reached. Moreover, only very few participants reached a subscore for the worst or best possible outcome, indicating no severe challenges with ceiling or floor effects.

## Discussion

We developed the TreatHSP-QoL, an HRQoL questionnaire specific for HSP patients that consists of 25 items divided into five subdomains: (I) general quality of life and attitude to the disease, (II) mobility and leisure time, (III) medical care, (IV) social life and occupation/work, and (V) associated symptoms. We also created a corresponding proxy questionnaire for the patients’ caregivers. Both questionnaires can be found in Additional file 5. The total score for TreatHSP-QoL ranges between 0 and 100, with higher values presenting a better HRQoL. The instructions and some examples for score calculation can be found in Additional file 6.

Given the low prevalence and heterogeneity of HSP, we were able to recruit a high number of patients and caregivers. We ensured that patients and caregivers were well represented throughout all project stages (Table 1). A total of 343 HSP patients and 127 caregivers participated overall. During the initial qualitative data acquisition, patients with both pure and complex HSP, different genotypes, and different disease severity (measured using the SPRS) were included. For the pilot validation, 65 patient and 53 caregiver responses out of 91 households were acquired, reflecting a high response rates of 71% and 58%, respectively. A solid sample size of 242 patient and 56 caregiver responses was obtained for the main validation. The ranges in sex, age, and SPRS during the pilot validation, and sex, age and disease duration during the main validation, demonstrates that the target population was represented at all times. For the PCOM development process, the unique perspective of patients and caregivers is essential. Accordingly, we obtained feedback on the interviews and the pilot-questionnaires. The development of other PCOMs for chronic neurological diseases may serve as a comparison. The development of the PDQ-39, a broadly used questionnaire for patients with Parkinson’ disease, was initially performed only on 227 patient responses [15].

To validate TreatHSP-QoL, extensive statistical tests and analyses were applied. The items were tested during the pilot validation for response rate, difficulty, inter-item correlations, corrected item-total correlations, and correlations to a general quality of life item. During the main validation, the structure of TreatHSP-QoL, with five subdomains that were identified by EFA, was confirmed by an acceptable fit of the CFA. The internal consistency was then measured via Cronbach’s alpha. Further test–retest correlations, as well as correlations with EQ-5D-5L and with FARS-ADL for all subdomains and for the total score, were explored. In addition to these statistical analyses, content-related aspects were considered. All of the TreatHSP-QoL subdomains are plausible in a clinical context. The subdomain with the lowest (i.e., most severe affection) HRQoL score on average was (II) mobility and leisure time, which is plausible since HSP is associated with lower limb spasticity and gait disorder, with consecutive mobility restrictions as main symptom in both pure and complicated HSP forms. The validation results did not show any significant impact of sex or age on the total score. While older age has been associated with lower HR-QoL values in general populations [27, 28], we did not necessarily expect this relation in HSP population. Firstly, younger individuals in general populations have less or none physical health problems compared to the older ones while all our participants already have HSP. Secondly, adaptation processes and coping strategies during the disease course in slowly progressive diseases like HSP may play an important role in HRQoL over the years. Even in general populations it was noticed, that subjective well-being or HRQoL values for mental health dimensions were stable or increasing with age despite physical decline, suggesting well adaptive behaviors and coping resources of the older individuals [29, 30]. In another study patients with specific chronic diseases also showed higher HRQoL values for mental components with increased age while those of physical components were poorer [31]. In terms of sex, there are different findings in general populations, mostly showing lower HRQoL values reported by females [28, 30, 32–37]. However also no significant impact of sex [38–41] or even higher HRQoL values for females have been reported [42].

Cronbach’s alpha was larger than 0.8 for three out of five subdomains and reached a total value of 0.85 for the whole questionnaire, which demonstrates good internal consistency (a value of 0.70 or above is considered acceptable for most research purposes [43–45]). The subdomain (V) associated symptoms reached a Cronbach’s alpha that was less than 0.6. It is not uncommon for HRQoL measures to be associated with lower Cronbach's alpha values due to the multidimensional nature of HRQoL [46]. In our case, it could be explained by the small number and diversity of the items belonging to this subdomain. However, all could be grouped together via EFA and were identified as important symptoms in HSP patients.

The positive correlations to EQ-5D-5L – an already well-validated HRQoL questionnaire were all significant except for the medical care subdomain, supporting the validity of TreatHSP-QoL. The highest value per subdomain of 0.52 and for the total score of 0.53 highlight the expected relevant difference between a generic and a disease specific HRQoL measure. The correlation with subdomain (III) medical care was close to 0 and not significant, as EQ-5D-5L does not address medical care issues. The good reliability of TreatHSP-QoL is proven by large test–retest correlations for all subdomains and for the total score, reaching a value of 0.86.

TreatHSP-QoL showed correlations between patient and their caregiver responses of > 0.7 for four out of five subdomains and a correlation of 0.65 for the total score. While there is no commonly accepted threshold for proxy-patient correlations, we claim that this result shows that the caregiver version of TreatHSP-QoL can be used as a valid proxy questionnaire. The lowest correlation of 0.64 was reached for the subdomain (I) general quality of life and attitude to the disease since this subdomain includes the most personal items like emotional wellbeing or concerns about the future. A meta-analysis of self- and proxy-reports of HRQoL in children also showed that the inter-rater agreement was lower for psychosocial-related domains such as emotion and cognition than for more observable domains relating to physical health and functioning [47].

Another aim that was addressed during the study was to explore the relation between HRQoL and clinical severity in HSP patients (i.e., FARS-ADL (a self-assessment disease severity measure where larger values represent higher severity) and the disease duration). For many chronic neurological diseases, studies have shown that HRQoL did not differ between diseased and healthy individuals [48, 49]. Even for fatal diseases like amyotrophic lateral sclerosis, it is not possible to predict whether HRQoL will differ between patients and healthy individuals [50]. This phenomenon is called the wellbeing paradox [51]. It also implies that the belief that neurodegenerative diseases are essentially associated with reduced HRQoL is incorrect. The correlation between the TreatHSP-QoL global score and the disease duration in years (considering more severe symptoms with longer disease duration due to progressive course of HSP) was close to 0. The lack of impact may be explained due to environmental adjustments and aids provided (e.g., increased mobility due to a wheelchair) or a better mental acceptance of disabilities that appear and progress slowly over many years. A suitable job and supporting environment may also play a role. TreatHSP-QoL and FARS-ADL were only slightly negatively correlated for the total score, and four out of five subdomains had absolute values lower than 0.5, thus supporting the wellbeing paradox. However, the subdomain (II) mobility and leisure time showed a relatively high inverse correlation of -0.59. This result is plausible in the clinical context of HSP and supports the validity of TreatHSP-QoL because FARS-ADL explores neurological symptoms such as gait and coordination problems and symptom-related limitations in daily activities and the items are similar to those of FARS-ADL. The correlations with other subdomains and the total score are rather low. This result is also plausible as these subdomains include items mostly covering aspects of HRQoL other than physical limitations and do not overlap with FARS-ADL-items. This also supports the expectation that HRQoL cannot simply be defined by the severity of clinical symptoms. Moreover, the correlation with the subdomain (III) medical care was close to 0, implying that disease severity and satisfaction with medical care are not necessarily linked. All together, these results show the importance of PCOM-assessment in general and emphasize the importance of using TreatHSP-QoL as an additional measure in both clinical trials and clinical practice, especially when evaluating therapies.

Finally, the high response rates of patients and caregivers during both the pilot and the main validation show the importance of PCOM-assessments to the patients themselves, and suggest that the assessment of TreatHSP-QoL may increase the motivation of HSP patients to participate in research studies. The German version of TreatHSP-QoL can also be completed via an online platform. This is convenient for many patients, as they do not have to travel, and simplifies the score calculation for researchers or clinicians. Using electronic tools enables easier collection of more cross sectional and longitudinal data.

Our project had several limitations. Although semi-structured, the interviews provided specific topics to consider and no additional qualitative data was acquired (e.g., social media, self-help groups, forums, etc.), which could cause a certain selection bias. This was counteracted by additional questions at the end of the interviews and open questions at the end of the pilot questionnaires. The EFA and CFA were conducted on the same sample because otherwise the data sets would have been too small. Only 31 corresponding patient and caregiver responses could be matched for the main validation, which might be considered a small sample size, especially compared to the total patient sample size. Further analyses of real-world data will be necessary to confirm the validity of the proxy questionnaire. No analyses of health problems other than HSP or the ones representing exclusion criteria (e.g., coxarthrosis, cancer) or certain life circumstances (e.g., death of a relative or a divorce) that could potentially affect HRQoL were performed. The reference to HSP was emphasized in every item; however, the influence of these additional circumstances on the total score can never be fully excluded. We have performed a translation-back translation process for TreatHSP-QoL, from German into English (i.e., the translation into English followed the back-translation method by translation to the target language by one translator and then back translation into German by a translator blinded to the original questionnaire) as a gold standard [52–54]; however, only the German version can be considered validated. Appropriate validation after translation to any other language is needed. Furthermore, no children took part so the use in pediatric patients is limited. Within the possible total score values between 0 and 100, it was not defined what difference value indicated a meaningful improvement or worsening of the HRQoL; in addition, there were no cut-off values for “good” or “poor” HRQoL. For accurate intra- and interindividual comparability of the score values, further real-world data validation is needed.

## Conclusion

TreatHSP-QoL can now be used as a disease-specific PCOM, i.e., HRQoL measure, for HSP patients, and is also available as a proxy questionnaire for caregivers. Overall, we demonstrated good validity and reliability. Our results highlight that HRQoL in general and TreatHSP-QoL in particular cannot be replaced by clinical scales, provide additional useful information, and should not be neglected in clinical trials or practice. TreatHSP-QoL could therefore be a meaningful additional outcome or an endpoint for high-standard treatment-effectiveness studies and precision health in HSP. It also may be used as a screening tool to choose the right patients at the right time to include them in clinical studies. In the growing field of genetic, but also symptomatic therapy studies, clinical trials would often have to run over a long period to determine a therapeutic effect, as the course of HSP is often slowly progressive. For trial readiness, it Is therefore essential to define primary and secondary endpoints that are specific to the disease and easy for patients to perform. The online platform, where TreatHSP-QoL can be completed, links the results directly to the German HSP registry (www.hsp-registry.net), following the need to establish PCOMs in general and TreatHSP-QoL in particular as a part of the patient registry data for natural course studies and other research projects. We believe that TreatHSP-QoL will serve as another piece of the mosaic to establish trial readiness for HSP in the future. In clinical practice, it is also a suitable tool for follow-up in a single patient, to add the patient’s perspective on disease progression or to evaluate therapies. As for every rare disease, international use of standardized measuring tools is highly useful in HSP. Translation and validation of TreatHSP-QoL into other languages are current projects pursued by the TreatHSP consortium.

## Methods

**Fig. 3:**
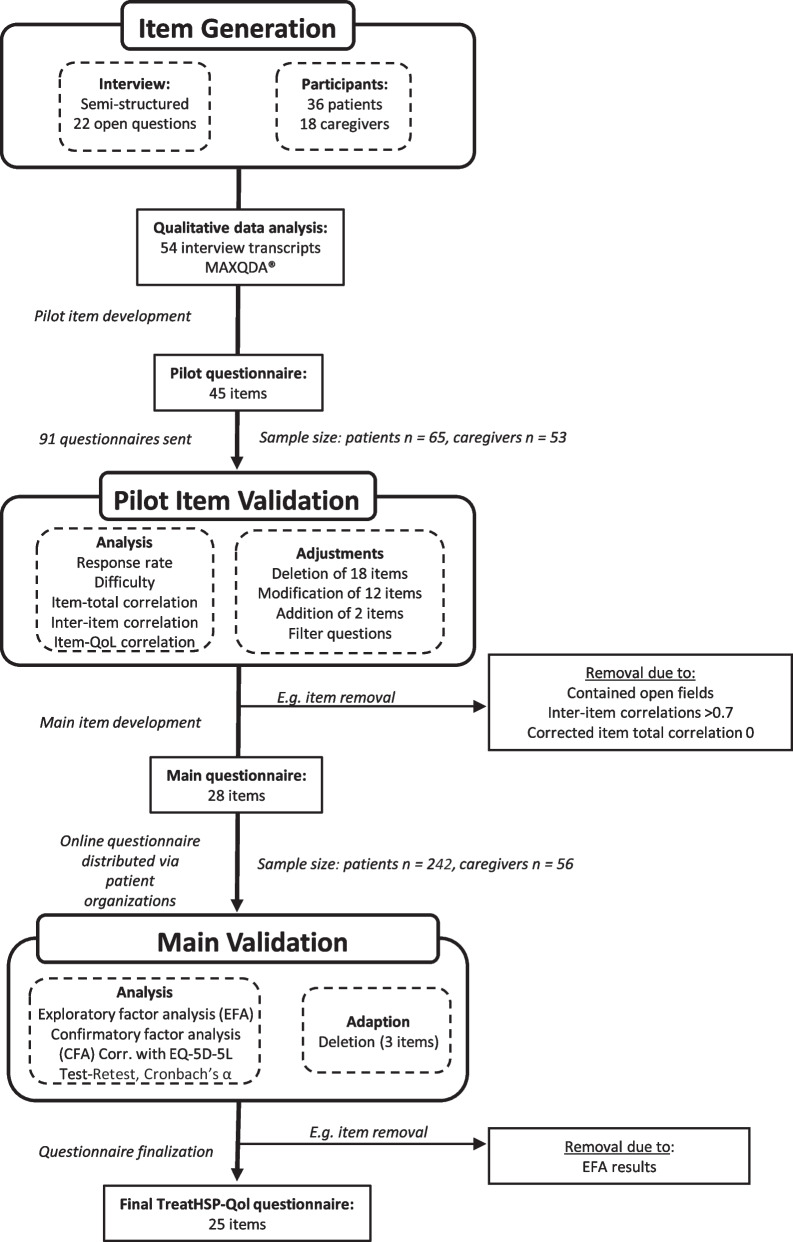
Overview of the TreatHSP-QoL development process. Three step approach with (I) pilot item generation relying on qualitative data analysis from the interviews, (II) pilot item validation on a small sample of patients and caregivers and (III) main validation on a larger sample of patients and caregivers. The figure also includes the aspects of development and modification shown in results section

Our aim was to develop and validate an HSP-specific HRQoL questionnaire, called TreatHSP-QoL. Accordingly, we chose a multistage approach based on grounded theory methodology and well-established, published PCOM development strategies [13, 55–57]. First, a large set of semi-structured interviews with patients and caregivers was used as a scaffold to develop a pilot version of a questionnaire. It was reviewed and revised in different subsequent steps. A modified questionnaire was then validated in a large group of patients and their caregivers and tested for their reliability and specificity for HSP (Fig. 3).

The interviews were conducted, video-recorded, and audio taped at the Department of Neurology, Essen University Hospital between August 2019 – January 2020. Due to the SARS-CoV-2 pandemic situation within this period, the participant recruitment was additionally challenging as all interviews were conducted in person. For the pilot item validation, printed questionnaires were sent via mail to HSP patients in the patient cohort of the Department of Neurology, University Hospital Essen. The main validation was performed using the online questionnaire tool (UmfrageOnline® (enuvo GmbH, Pfäffikon, Switzerland)).

### Step 1: item generation for pilot questionnaire

Conception of the pilot items was based on the basic principles of PCOM development, i.e., active participation of the patients and systematic data collection. To identify the main aspects of the disease that affect patients’ daily lives from their own perspective, data were collected using semi-structured interviews. The interview was designed based on general and clinical knowledge about HSP symptoms and its natural course. It contained guiding open questions that covered different aspects of daily life and focused on subjective perceptions of patients, e.g., about symptom-related limitations, their impact on leisure activities, relationships, occupation and personal care, and about treatments received. Furthermore, emotional wellbeing and attitude toward the disease and associated disabilities were explored. After finishing the open questions, the participants were asked for free associations to 12 terms (e.g., quality of life, future, aids, etc.). Finally, participants were given the opportunity to give feedback on comprehensibility and relevance of the questions asked and to add information on topics they considered relevant and that had not been mentioned during the interview (the interview guide, translated into English for illustrative purposes, can be found in Additional file 7). No alternative interview was designed for caregivers, as they were asked the same questions as the patients, with the instruction to answer them from the patients’ perspective (i.e., proxy questionnaire approach). To allow in-depth concept exploration, we chose to perform one-to-one interviews rather than focus-group interviews. No data was available that could be used to calculate the sample size to achieve qualitative data saturation for HSP. Therefore, we choose an empirical approach supported by the literature with 35 patient and 35 caregiver interviews [58, 59]. The participants were recruited out of the HSP-patient cohort in the Department of Neurology, University Hospital Essen and via HSP patient organizations (see acknowledgements). The inclusion criteria for patients were a confirmed clinical and/or genetic diagnosis of HSP and the age of ≥ 16 years. Exclusion criteria besides the age below 16 years was a co-morbidity with another chronic and severe disease that is likely to impact the quality of life or with a neurodegenerative disease or movement disorder other than HSP (e.g., Parkinson’s disease). Patients unable to give informed consent due to cognitive impairment were enrolled using consent of their legal representatives and were engaged to complete the interviews to the best of their ability, being interviewed together with their proxies. Not affected family members (first or second-degree relatives) and caregivers ≥ 18-year-old were included as proxies. To ensure all disease severity degrees were represented patients were examined at the Department of Neurology, University Hospital Essen. Therefore, the ability to participate for the interviews in person was a prerequisite. The patients were selected and classified according to their total SPRS score as follows: mildly affected (SPRS 0–15 points), moderately affected (SPRS 16–30 points), severely affected (SPRS 31–52 points). Caregivers were also grouped according to the total SPRS score of their affected relatives.

For qualitative data analysis thematic analysis of the interview transcripts (Microsoft Word®, Microsoft, Washington, USA) was applied to generate (sub)domains (codes), matrix coding was then performed using a commercially available qualitative and mixed-methods research software (MAXQDA®, VERBI Software GmbH, Berlin, Germany). Analyzing patterns within the matrix enabled pilot items to be created. The frequently repeated statements of the respondents were implemented in the item wording. The generated items were compiled into separate pilot questionnaires for patients and their caregivers. Additionally, at the end of the questionnaires, open feedback from the participants was collected.

The patient and caregiver pilot questionnaires, containing 45 items each, were discussed with German clinician experts from the TreatHSP consortium to ensure that their content was in agreement with expert knowledge about the disease, and their wording was understandable.

### Step 2: pilot item validation

The pilot questionnaires were sent by mail to households from the HSP patient cohort in the Department of Neurology, University Hospital Essen. No personal or medical data were additionally obtained from participants, and responses were anonymized. The items were five-point Likert items. For validation, they were given values between zero and four. The total score was calculated by summing up all item values with a maximum score of 180. Response rate, difficulty (i.e., the average item result divided by the maximal/worst possible result), standard deviation, inter-item correlations, and corrected item-total correlations were analyzed. All correlations were estimated using the sample Pearson correlation coefficient. Pilot item validation could be performed on a smaller sample size (e.g., ≤ 100 respondents) [60]. Considering the difficulty of recruiting patients with a rare disease a sample size large enough for precise estimation of 50 patients and 50 caregivers was aimed. The items were then reduced or improved according to the results, e.g., all items with inter-item correlations above 0.7 or corrected item-total correlations smaller than 0.3 were considered for removal [60]. Furthermore, the content and the wording were critically reviewed based on feedback from the participants at the end of the questionnaires.

### Step 3: main validation

The modified questionnaire, containing 28 items, was implemented online (UmfrageOnline®, enuvo GmbH, Pfäffikon, Switzerland) in a patient and a caregiver version (as a proxy questionnaire). First, it was presented to representatives of the German HSP patient organizations to ensure comprehensibility and technical accuracy, then distributed among HSP patient organization members in three German-speaking countries: Germany, Austria, and Switzerland. The participation was pseudonymous, the patient demographic data obtained were sex and year of birth (in both the patient and caregiver questionnaires). As we were unable to access any reliable medical data considering disease severity, participants were requested to fill out the German version of Friedreich Ataxia Rating Scale-Activities of Daily Living (FARS-ADL) – a self-completion questionnaire of the disease and symptom severity, originally developed and well-validated for Friedreich ataxia patients [61, 62]. This scale was chosen because Friedreich’s ataxia shows similarities to HSP (due to a mainly slowly progressive gait disorder and concomitant neurological symptoms such as speech disorder or urge incontinence as well as disease heterogeneity) and no such HSP-specific self-assessment instrument currently exists. Finally, participants were asked to complete the German version of the EuroQol-5 Dimension (5 levels) questionnaire (EQ-5D-5L), which is a well-validated, generic HRQoL questionnaire [63].

In the main validation phase, an exploratory factor analysis (EFA) was conducted to detect the underlying structure of the questionnaire and define its subdomains, with principal component analysis and varimax rotation. It was checked whether the data set was suitable for factor analysis using the Kaiser–Meyer–Olkin (KMO) criterion. The KMO is a measure that can reach values between zero and one, where a value close to one indicates that the data is adequate for EFA. To determine the number of factors, i.e., the number of subdomains, only factors with an eigenvalue greater than one, i.e., only factors that account for more variance than a single item, were considered. Additionally, the scree plot was taken into account to determine the number of factors. The scree plot considers the eigenvalue of each factor and thus the additional percentage of explained variance per factor. Only items with factor loadings of at least 0.4 were considered for further analyses. Items with the main factor loading of less than 0.5 and a difference to other factors of 0.15 or less were critically reviewed. With respect to their content, it was then decided whether these items should remain in the final questionnaire or not. The identified structure was then cross-checked using confirmatory factor analysis (CFA). The CFI, the RMSEA, and the SRMR were used to judge the model. The CFI, RMSEA and SRMR can reach values between zero and one, where a value close to one for the CFI and values close to zero for RMSEA and SRMR indicate a good model fit. The CFA was conducted on the same data as the EFA, since sample size was considered too small to conduct separate analyses.

For the EFA it was aimed to achieve a minimum sample size of 10 patient respondents per item, but a minimum of 200 respondents [64]. It was planned to conduct CFA on an independent data set, if at least 100 additional respondents to the minimum number for EFA could be achieved [65].

The final items, that passed the criteria mentioned above, remained the five-point Likert items, with values between zero and four. The questionnaire scores, total or per subdomain, were calculated by the mean value of all answered items multiplied by 25, so that an HRQoL score between 0 and 100 could be reached (0 = the worst possible HRQoL, 100 = the best possible HRQoL in terms of this questionnaire). More details about the score calculation and some examples can be found in the Additional file 6. To determine convergent validity of the final questionnaire, correlations with the EQ-5D-5L and FARS-ADL scores were calculated for subdomains and in total. Correlations between patient and their caregiver scores were evaluated for subdomains and in total using the sample Pearson’s correlation coefficient. To test reliability, the questionnaires were re-sent two weeks after they were completed to the participants who had agreed to be available for a retest. In addition, Cronbach’s alpha was calculated per subdomain and in total.

Statistical analyses in the pilot and the main validation were conducted using analytics software (SAS software, version 9.4. Statistical Analysis System, SAS Institute Inc., North Carolina, USA). Graphics were generated using R, version 4.1.1 [66] and SAS software.

### Supplementary Information

**Additional file 1:** Pilot item validation.

**Additional file 2:** Total replies and replies that were used for the main validation for the patient and caregiver questionnaires as well as for the retest of the patient questionnaire.

**Additional file 3:** Factor loadings, which were obtained by exploratory factor analysis with the final grouping of items, where dark red represents deleted items, light green grouped items, and light orange a small main factor loading and/or a small difference to the second largest factor loading of the item.

**Additional file 4:** **Table S1.** Distribution of the subscores and total score for the patient self-reported questionnaire. **Table S2.** Distribution of the subscores and total score for the caregiver-reported questionnaire.

**Additional file 5:** TreatHSP-QoL: Final HRQoL questionnaire for patients, translated here from German to English for illustrative purposes. TreatHSP-QoL: Final HRQoL Questionnaire for caregivers, translated here from German to English for illustrative purposes.

**Additional file 6:** Score calculation for TreatHSP-QoL.

**Additional file 7:** Interview guide, translated here from German to English for illustrative purposes.

## Data Availability

The datasets generated and/or analyzed during the current study are not publicly available due to the privacy policy but are available from the corresponding author on reasonable request.

## Abbreviations

CFA: Confirmatory factor analysis
CFI: Comparative fit index
EFA: Exploratory factor analysis
EQ-5D-5L: EuroQol-5 dimension (5 levels)
FARS-ADL: Friedreich ataxia rating scale-activities of daily living
HRQoL: Health-related quality of life
HSP: Hereditary spastic paraplegia
PCOM: Patient-centered outcome measure
RMSEA: Root mean square error of approximation
SPG: Spastic paraplegia gene
SPRS: Spastic paraplegia rating scale
SRMSR: Standardized root mean square residual
