# Supplementary material for: Development and validation of TreatHSP-QoL: a patient-reported outcome measure for health-related quality of life in hereditary spastic paraplegia
Source: Orphanet J Rare Dis. 2024 Jan 2;19:2. doi: 10.1186/s13023-023-03012-w (PMC10763482; doi:10.1186/s13023-023-03012-w)
Supplement: Supplementary file 1 — Additional file 1: Pilot item validation. [file 13023_2023_3012_MOESM1_ESM.docx]

**Additional file 1. Pilot item validation**

Out of patients’ responses single items were analysed for response rate, inter-item correlations, item-total correlation, and correlation to the item on general quality of life (PGE01_A1). The heatmap with the results can be found in Figure 1. Based on the patients’ responses, four items were first deleted because inter-item correlations were above 0.69, indicating very similar items (items PSY02_A2, PRE02_A3, PRE03_A1, PSO04_A1). Six additional items were deleted because their content was similar to others’ or they could be combined with other items (items PEH02_A1, PEH04_A1, PSO02_A1, PEM03_A1, PDI02_A1, PDI04_A1). One item was deleted because it did not correlate with the sum of all items apart from the item itself, with a corrected item-total correlation equal to 0.01 (item PRE02_A4). One item was deleted because it had a very small correlation to the item on general quality of life with a value of 0.04 (item PSO06_A1). Three additional items were deleted for content reasons in respect to comprehensibility, applicability and further evaluation of the questionnaire (items PMO03_A1, PME04_A1, PME06_A1). Finally, the three items with free text fields that were initially added to capture additional symptoms were also deleted (items PSY02_A7, PSY02_A8, PSY03_A9).

Most of the remaining items had a response rate of at least 97%, meaning that each item was answered by at least 63 out of 65 patients. However, there were two items that had low response rates (regarding usable answers) of 62% and 57% (items PEM01_A1, PEM02_A1). These were items regarding work or occupation. These items were modified such that they are now preceded by filter questions that ask whether the patient is working and, if the patient is not working, whether this is due to the HSP. If the patient is working, they are led to an item, asking whether working is possible without any restrictions. If the patient is not working due to the HSP, they are led to an item, asking whether the lack of work has a negative impact. This means that these two items are count for one in terms of this questionnaire, as each patient only can answer one of them. If the patient is not working for other reason than HSP, no further item has to be answered. Additional filter questions were also added for two other items, asking, whether a walking aid or other people’s help is needed, however, without modifying the original items (items PMO02_A1 and PEH01_A1).

The symptom items with open fields revealed that the following symptoms in particular were missing for some patients: intestinal complaints, headaches/migraine, lower limb complaints (in the pilot items these were only addressed indirectly, e.g. via items on mobility, balance, cramps etc.), and difficulties in concentrating. Accordingly, two new symptom items were added, one for the lower limb and one for difficulties in concentrating. To include intestinal complaints, the item originally asking for bladder disorder (item PSY02_A3) was modified. The item originally asking for pain (item PSY02_A4) was modified to include headache. Ten additional items were modified, mainly to include aspects of strongly correlated deleted items, but also to improve wording.

Thus, in total, out of the 45 pilot items 18 items were deleted, 12 items were modified, and two new items were added. Filter questions were added prior to some items to improve comprehensibility. The filter questions about work/occupational situation led from formal 29 items per questionnaire to actual 28 items per participant for the main validation.
