# Supplementary material for: Development and validation of TreatHSP-QoL: a patient-reported outcome measure for health-related quality of life in hereditary spastic paraplegia
Source: Orphanet J Rare Dis. 2024 Jan 2;19:2. doi: 10.1186/s13023-023-03012-w (PMC10763482; doi:10.1186/s13023-023-03012-w)
Supplement: Supplementary file 2 — Additional file 2: Total replies and replies that were used for the main validation for the patient and caregiver questionnaires as well as for the retest of the patient questionnaire. [file 13023_2023_3012_MOESM2_ESM.docx]

**Additional file 2.** Total replies and replies that were used for the main validation for the patient and caregiver questionnaires as well as for the retest of the patient questionnaire.

|  | Patients | | Caregivers | | Retest | |
| --- | --- | --- | --- | --- | --- | --- |
|  | n | % | n | % | n | % |
| Total number of replies | 315 | 100 | 91 | 100 | 104 | 100 |
| Number of HRQoL not completed | 67 | 21 | 26 | 29 | 8 | 8 |
| Number of duplicates | 3 | 1 | 3 | 3 | 0 | 0 |
| Number of patients aged ≤16 years | 3 | 1 | 6 | 7 | 0 | 0 |
| Number of HRQoL replies for analysis | 242 | 77 | 56 | 62 | 96 | 92 |

HRQoL, health-related quality of life
