# Supplementary material for: Development and validation of TreatHSP-QoL: a patient-reported outcome measure for health-related quality of life in hereditary spastic paraplegia
Source: Orphanet J Rare Dis. 2024 Jan 2;19:2. doi: 10.1186/s13023-023-03012-w (PMC10763482; doi:10.1186/s13023-023-03012-w)
Supplement: Supplementary file 3 — Additional file 3: Factor loadings, which were obtained by exploratory factor analysis with the final grouping of items, where dark red represents deleted items, light green grouped items, and light orange a small main factor loading and/or a small difference to the second largest factor loading of the item. [file 13023_2023_3012_MOESM3_ESM.docx]

**Additional file 3****.** Factor loadings, which were obtained by exploratory factor analysis with the final grouping of items, where dark red represents deleted items, light green grouped items, and light orange a small main factor loading and/or a small difference to the second largest factor loading of the item.

|  | Factor 1 | Factor 2 | Factor 3 | Factor 4 | Factor 5 | Factor Max | Difference |
| --- | --- | --- | --- | --- | --- | --- | --- |
| PDA03 | 0,80 | 0,02 | 0,13 | 0,10 | 0,19 | 1 | 0.61 |
| PDA01 | 0.74 | 0.04 | 0.28 | -0.03 | 0.25 | 1 | 0.45 |
| PDA02 | 0.72 | -0.13 | 0.14 | 0.18 | 0.12 | 1 | 0.54 |
| PSY01 | 0.64 | 0.32 | 0.15 | 0.07 | 0.19 | 1 | 0.32 |
| PRE01 | 0.63 | 0.27 | 0.13 | 0.24 | -0.07 | 1 | 0.35 |
| PQL01 | 0.62 | 0.31 | 0.18 | 0.13 | 0.01 | 1 | 0.30 |
| PSO01 | 0.49 | 0.27 | 0.13 | 0.34 | -0.01 | 1 | 0.15 |
| PRE03 | 0.12 | 0.77 | -0.14 | 0.05 | -0.10 | 2 | 0.65 |
| PMO01 | 0.30 | 0.76 | -0.05 | 0.06 | -0.13 | 2 | 0.46 |
| PSY02_1 | 0.10 | 0.74 | 0.04 | -0.01 | 0.04 | 2 | 0.64 |
| PSY02_2 | -0.10 | 0.61 | 0.03 | 0.08 | 0.14 | 2 | 0.47 |
| PSY02_6 | 0.10 | 0.56 | 0.11 | 0.00 | 0.40 | 2 | 0.16 |
| PRE02 | 0.41 | 0.54 | -0.03 | 0.19 | -0.24 | 2 | 0.13 |
| PMO02_PMO03 | 0.14 | 0.41 | 0.30 | 0.26 | 0.11 | 2 | 0.11 |
| PHE01_PHE02 | 0.21 | 0.37 | 0.04 | 0.34 | 0.24 | 2 | 0.04 |
| PMD02 | 0.12 | -0.06 | 0.85 | 0.03 | -0.15 | 3 | 0.73 |
| PMD03 | 0.21 | -0.05 | 0.80 | 0.01 | 0.10 | 3 | 0.59 |
| PMD01 | 0.23 | 0.07 | 0.78 | 0.01 | 0.04 | 3 | 0.55 |
| PMD04 | 0.39 | 0.16 | 0.53 | 0.14 | -0.13 | 3 | 0.15 |
| PSO02 | 0.15 | 0.07 | 0.07 | 0.73 | -0.03 | 4 | 0.57 |
| PEF05 | 0.16 | 0.21 | -0.13 | 0.56 | 0.25 | 4 | 0.31 |
| PHE03 | -0.01 | -0.19 | 0.21 | 0.55 | -0.01 | 4 | 0.34 |
| PEF03_PEF04 | 0.13 | 0.10 | -0.18 | 0.54 | 0.17 | 4 | 0.37 |
| PSO03 | 0.23 | 0.22 | 0.25 | 0.50 | 0.21 | 4 | 0.25 |
| PSY02_5 | -0.05 | 0.12 | 0.09 | 0.21 | 0.72 | 5 | 0.50 |
| PSY02_7 | 0.15 | 0.02 | -0.11 | 0.14 | 0.67 | 5 | 0.52 |
| PSY02_4 | 0.12 | -0.10 | -0.08 | 0.02 | 0.51 | 5 | 0.39 |
| PSY02_3 | 0.14 | 0.42 | 0.15 | 0.02 | 0.46 | 5 | 0.04 |
