# Supplementary material for: Development and validation of TreatHSP-QoL: a patient-reported outcome measure for health-related quality of life in hereditary spastic paraplegia
Source: Orphanet J Rare Dis. 2024 Jan 2;19:2. doi: 10.1186/s13023-023-03012-w (PMC10763482; doi:10.1186/s13023-023-03012-w)
Supplement: Supplementary file 4 — Additional file 4: Table S1. Distribution of the subscores and total score for the patient self-reported questionnaire. Table S2. Distribution of the subscores and total score for the caregiver-reported questionnaire. [file 13023_2023_3012_MOESM4_ESM.docx]

**Additional file 4.**

**Table 1.** Distribution of the subscores and total score for the patient self-reported questionnaire.

|  | **Min** | **Q1** | **Mean** | **Median** | **Q3** | **Max** | **SD** |
| --- | --- | --- | --- | --- | --- | --- | --- |
| General QoL and attitude to disease | 0.0 | 37.5 | 52.4 | 54.2 | 66.7 | 100.0 | 19.3 |
| Mobility and leisure time | 0.0 | 15.0 | 31.4 | 30.0 | 45.0 | 95.0 | 20.4 |
| Medical care | 6.3 | 50.0 | 64.1 | 62.5 | 75.0 | 100.0 | 21.1 |
| Social life and occupation/work | 12.5 | 55.0 | 65.7 | 65.0 | 80.0 | 100.0 | 19.7 |
| Associated symptoms | 12.5 | 68.8 | 79.1 | 81.3 | 93.8 | 100.0 | 16.3 |
| Total score | 25.0 | 48.0 | 56.6 | 57.0 | 64.0 | 92.0 | 13.0 |

Max, maximum; Min, minimum; Q1, first quantile; Q3, third quantile; QoL, quality of life; SD, standard deviation.

**Table 2.** Distribution of the subscores and total score for the caregiver-reported questionnaire.

|  | **Min** | **Q1** | **Mean** | **Median** | **Q3** | **Max** | **SD** |
| --- | --- | --- | --- | --- | --- | --- | --- |
| General QoL and attitude to disease | 0.0 | 35.4 | 47.1 | 45.8 | 58.3 | 83.3 | 19.5 |
| Mobility and leisure time | 0.0 | 20.8 | 29.4 | 27.1 | 39.6 | 79.2 | 17.3 |
| Medical care | 12.5 | 50.0 | 60.8 | 62.5 | 75.0 | 93.8 | 18.8 |
| Social life and occupation/work | 5.0 | 45.0 | 55.9 | 60.0 | 70.0 | 95.0 | 20.7 |
| Associated symptoms | 6.3 | 68.8 | 75.1 | 75.0 | 87.5 | 100.0 | 19.6 |
| Total score | 10.0 | 45.0 | 51.3 | 52.5 | 61.0 | 80.0 | 13.5 |

Max, maximum; Min, minimum; Q1, first quantile; Q3, third quantile; QoL, quality of life; SD, standard deviation.
