# Supplementary material for: Development and validation of TreatHSP-QoL: a patient-reported outcome measure for health-related quality of life in hereditary spastic paraplegia
Source: Orphanet J Rare Dis. 2024 Jan 2;19:2. doi: 10.1186/s13023-023-03012-w (PMC10763482; doi:10.1186/s13023-023-03012-w)
Supplement: Supplementary file 5 — Additional file 5: TreatHSP-QoL: Final HRQoL questionnaire for patients, translated here from German to English for illustrative purposes. TreatHSP-QoL: Final HRQoL Questionnaire for caregivers, translated here from German to English for illustrative purposes. [file 13023_2023_3012_MOESM5_ESM.docx]

**Additional file 5.**

**TreatHSP-QoL:** Final HRQoL questionnaire for patients, translated here from German to English for illustrative purposes.

# (I) General quality of life and attitude to the disease

**Please indicate how accurate the following statement is.**

|  | Strongly disagree | Disagree | Partially agree | Agree | Strongly agree |
| --- | --- | --- | --- | --- | --- |
| 1. I am satisfied with my overall quality of life. | □󠆯 | □󠆯 | □󠆯 | □󠆯 | □󠆯 |
| 2. I cope well with my symptoms. | □󠆯 | □󠆯 | □󠆯 | □󠆯 | □󠆯 |
| 3. Overall, I am satisfied with my leisure activities. | □󠆯 | □󠆯 | □󠆯 | □󠆯 | □󠆯 |
| 4. Personally, I deal with my disease appropriately. | □󠆯 | □󠆯 | □󠆯 | □󠆯 | □󠆯 |
| 5. I am afraid of the future (e.g. dependence on a wheelchair, financial losses, partnership/social life etc.) because I do not know how my disease will progress. | □󠆯 | □󠆯 | □󠆯 | □󠆯 | □󠆯 |

**Please indicate how often the following statement is true.**

|  | Never | Rarely | Sometimes | Frequently | Very frequently |
| --- | --- | --- | --- | --- | --- |
| 6. When thinking about my disease, it triggers negative emotions/a depressed mood in me. | □󠆯 | □󠆯 | □󠆯 | □󠆯 | □󠆯 |

# (II) Mobility and leisure time

**To what extent do you feel restricted in your daily life by the following symptoms associated with HSP?**

|  | Not present / Not restricted at all | Slightly restricted | Moderately restricted | Very restricted | Extremely restricted |
| --- | --- | --- | --- | --- | --- |
| 7. Restricted mobility (incl. gait and balance disorders) | □󠆯 | □󠆯 | □󠆯 | □󠆯 | □󠆯 |
| 8. Disorders of the bladder or gastrointestinal tract | □󠆯 | □󠆯 | □󠆯 | □󠆯 | □󠆯 |
| 9. Lower limb complaints (e.g. trembling, cramps, lack of strength, sensitivity) | □󠆯 | □󠆯 | □󠆯 | □󠆯 | □󠆯 |

**Please indicate how accurate the following statement is.**

|  | Strongly disagree | Disagree | Partially agree | Agree | Strongly agree |
| --- | --- | --- | --- | --- | --- |
| 10. I feel restricted in my mobility due to my disease. | □󠆯 | □󠆯 | □󠆯 | □󠆯 | □󠆯 |
| 11. Due to my disease, I am **not able** to do everything I would like to do in my free time. | □󠆯 | □󠆯 | □󠆯 | □󠆯 | □󠆯 |
| 12. Due to my disease, my leisure activities require special planning in advance (e.g. information about accessibility, toilets, general accessibility of the venue). | □󠆯 | □󠆯 | □󠆯 | □󠆯 | □󠆯 |

# (III) Medical care

**Please indicate how accurate the following statement is.**

|  | Strongly disagree | Disagree | Partially agree | Agree | Strongly agree |
| --- | --- | --- | --- | --- | --- |
| 13. Overall, I am satisfied with my doctors or therapists. | □󠆯 | □󠆯 | □󠆯 | □󠆯 | □󠆯 |
| 14. I have an expert contact person for questions about my disease. | □󠆯 | □󠆯 | □󠆯 | □󠆯 | □󠆯 |
| 15. I am satisfied with my level of information about my disease. | □󠆯 | □󠆯 | □󠆯 | □󠆯 | □󠆯 |
| 16. My therapies /medications make me feel better. | □󠆯 | □󠆯 | □󠆯 | □󠆯 | □󠆯 |

# (IV) Social life and occupation/work

**Please indicate how accurate the following statement is.**

|  | Strongly disagree | Disagree | Partially agree | Agree | Strongly agree |
| --- | --- | --- | --- | --- | --- |
| 17. My daily life is organised appropriately in accordance with my disease (e.g. home help, nursing service, transport service, support from friends and/or family). | □󠆯 | □󠆯 | □󠆯 | □󠆯 | □󠆯 |
| 18. Due to my disease, I experience exclusion from people who are close to me. | □󠆯 | □󠆯 | □󠆯 | □󠆯 | □󠆯 |
| 19. I experience stigmatisation due to my disease (e.g. because of my striking gait). | □󠆯 | □󠆯 | □󠆯 | □󠆯 | □󠆯 |

**Are you currently employed?**

Employment is understood to be any paid activity or activity that is associated with an income, regardless of the amount of time it takes.

□󠆯 Yes → Please proceed to the statement 20.1. and skip the statement 20.2.

□󠆯 No → Please proceed to the next question.

**Are you not (or no longer) employed due to your disease?**

□󠆯 Yes → Please proceed to the statement 20.2.

□󠆯 No → Please proceed to the statement 21.

**Please indicate how accurate the following statement is.**

|  | Strongly disagree | Disagree | Partially agree | Agree | Strongly agree |
| --- | --- | --- | --- | --- | --- |
| 20.1. I can do my job without restrictions. | □󠆯 | □󠆯 | □󠆯 | □󠆯 | □󠆯 |

**Please indicate how accurate the following statement is.**

|  | Strongly disagree | Disagree | Partially agree | Agree | Strongly agree |
| --- | --- | --- | --- | --- | --- |
| 20.2. I find the lack of employment a burden. | □󠆯 | □󠆯 | □󠆯 | □󠆯 | □󠆯 |

**Please indicate how accurate the following statement is.**

|  | Strongly disagree | Disagree | Partially agree | Agree | Strongly agree |
| --- | --- | --- | --- | --- | --- |
| 21. My financial situation has deteriorated due to my disease or its treatment. | □󠆯 | □󠆯 | □󠆯 | □󠆯 | □󠆯 |

# (V) Associated symptoms

**To what extent do you feel restricted in your daily life by the following symptoms associated with HSP?**

|  | Not present / Not restricted at all | Slightly restricted | Moderately restricted | Very restricted | Extremely restricted |
| --- | --- | --- | --- | --- | --- |
| 22. Pain in general (e.g. headache, limb pain, muscle pain) | □󠆯 | □󠆯 | □󠆯 | □󠆯 | □󠆯 |
| 23. Speech disorders | □󠆯 | □󠆯 | □󠆯 | □󠆯 | □󠆯 |
| 24. Upper limb complaints (e.g. trembling, cramps, lack of strength, sensitivity) | □󠆯 | □󠆯 | □󠆯 | □󠆯 | □󠆯 |
| 25. Memory /concentration disorders | □󠆯 | □󠆯 | □󠆯 | □󠆯 | □󠆯 |

**TreatHSP-QoL:** Final HRQoL Questionnaire for caregivers, translated here from German to English for illustrative purposes.

# (I) General quality of life and attitude to the disease

**Please indicate how accurate the following statement is.**

|  | Strongly disagree | Disagree | Partially agree | Agree | Strongly agree |
| --- | --- | --- | --- | --- | --- |
| 1. My relative is satisfied with his/her overall quality of life. | □󠆯 | □󠆯 | □󠆯 | □󠆯 | □󠆯 |
| 2. My relative copes well with his/her symptoms. | □󠆯 | □󠆯 | □󠆯 | □󠆯 | □󠆯 |
| 3. Overall, my relative is satisfied with his/her leisure activities. | □󠆯 | □󠆯 | □󠆯 | □󠆯 | □󠆯 |
| 4. My relative deals with his/her disease appropriately. | □󠆯 | □󠆯 | □󠆯 | □󠆯 | □󠆯 |
| 5. My relative is afraid of the future (e.g. dependence on a wheelchair, financial losses, partnership/social life etc.) because he/she does not know how his/her disease will progress. | □󠆯 | □󠆯 | □󠆯 | □󠆯 | □󠆯 |

**Please indicate how often the following statement is true.**

|  | Never | Rarely | Sometimes | Frequently | Very frequently |
| --- | --- | --- | --- | --- | --- |
| 6. Due to the disease and associated limitations, my relative has a depressed mood. | □󠆯 | □󠆯 | □󠆯 | □󠆯 | □󠆯 |

# (II) Mobility and leisure time

**To what extent does your relative feel restricted in his/her daily life by the following symptoms associated with HSP?**

|  | Not present / Not restricted at all | Slightly restricted | Moderately restricted | Very restricted | Extremely restricted |
| --- | --- | --- | --- | --- | --- |
| 7. Restricted mobility (incl. gait and balance disorders) | □󠆯 | □󠆯 | □󠆯 | □󠆯 | □󠆯 |
| 8. Disorders of the bladder or gastrointestinal tract | □󠆯 | □󠆯 | □󠆯 | □󠆯 | □󠆯 |
| 9. Lower limb complaints (e.g. trembling, cramps, lack of strength, sensitivity) | □󠆯 | □󠆯 | □󠆯 | □󠆯 | □󠆯 |

**Please indicate how accurate the following statement is.**

|  | Strongly disagree | Disagree | Partially agree | Agree | Strongly agree |
| --- | --- | --- | --- | --- | --- |
| 10. My relative feels restricted in his/her mobility due to the disease. | □󠆯 | □󠆯 | □󠆯 | □󠆯 | □󠆯 |
| 11. Due to the disease, my relative is **unable** to do everything he/she would like to do in his/her free time. | □󠆯 | □󠆯 | □󠆯 | □󠆯 | □󠆯 |
| 12. Due to the disease, my relative’s leisure activities require special planning in advance (e.g. information about accessibility, toilets, general accessibility of the venue). | □󠆯 | □󠆯 | □󠆯 | □󠆯 | □󠆯 |

# (III) Medical care

**Please indicate how accurate the following statement is.**

|  | Strongly disagree | Disagree | Partially agree | Agree | Strongly agree |
| --- | --- | --- | --- | --- | --- |
| 13. Overall, my relative is satisfied with his/her doctors or therapists. | □󠆯 | □󠆯 | □󠆯 | □󠆯 | □󠆯 |
| 14. My relative has an expert contact person for questions about the disease. | □󠆯 | □󠆯 | □󠆯 | □󠆯 | □󠆯 |
| 15. My relative is satisfied with his/her level of information about the disease. | □󠆯 | □󠆯 | □󠆯 | □󠆯 | □󠆯 |
| 16. My relative’s therapies /medications make him/her feel better. | □󠆯 | □󠆯 | □󠆯 | □󠆯 | □󠆯 |

# (IV) Social life and occupation/work

**Please indicate how accurate the following statement is.**

|  | Strongly disagree | Disagree | Partially agree | Agree | Strongly agree |
| --- | --- | --- | --- | --- | --- |
| 17. My relative’s daily life is organised appropriately in accordance with the disease (e.g. home help, nursing service, transport service, support from friends and/or family). | □󠆯 | □󠆯 | □󠆯 | □󠆯 | □󠆯 |
| 18. Due to the disease, my relative has withdrawn socially. | □󠆯 | □󠆯 | □󠆯 | □󠆯 | □󠆯 |
| 19. My relative experiences stigmatisation due to the disease (e.g. because of a striking gait). | □󠆯 | □󠆯 | □󠆯 | □󠆯 | □󠆯 |

**Is your relative currently employed?**

Employment is understood to be any paid activity or activity that is associated with an income, regardless of the amount of time it takes.

□󠆯 Yes → Please proceed to the statement 20.1. and skip the statement 20.2.

□󠆯 No → Please proceed to the next question.

**Is your relative not (or no longer) employed due to your disease?**

□󠆯 Yes → Please proceed to the statement 20.2.

□󠆯 No → Please proceed to the statement 21.

**Please indicate how accurate the following statement is.**

|  | Strongly disagree | Disagree | Partially agree | Agree | Strongly agree |
| --- | --- | --- | --- | --- | --- |
| 20.1. My relative can do his/her job without restrictions. | □󠆯 | □󠆯 | □󠆯 | □󠆯 | □󠆯 |

**Please indicate how accurate the following statement is.**

|  | Strongly disagree | Disagree | Partially agree | Agree | Strongly agree |
| --- | --- | --- | --- | --- | --- |
| 20.2. My relative finds the lack of employment a burden. | □󠆯 | □󠆯 | □󠆯 | □󠆯 | □󠆯 |

**Please indicate how accurate the following statement is.**

|  | Strongly disagree | Disagree | Partially agree | Agree | Strongly agree |
| --- | --- | --- | --- | --- | --- |
| 21. My relative’s financial situation has deteriorated due to the disease or its treatment. | □󠆯 | □󠆯 | □󠆯 | □󠆯 | □󠆯 |

# (V) Associated symptoms

**To what extent does your relative feel restricted in his/her daily life by the following symptoms associated with HSP?**

|  | Not present / Not restricted at all | Slightly restricted | Moderately restricted | Very restricted | Extremely restricted |
| --- | --- | --- | --- | --- | --- |
| 22. Pain in general (e.g. headache, limb pain, muscle pain) | □󠆯 | □󠆯 | □󠆯 | □󠆯 | □󠆯 |
| 23. Speech disorders | □󠆯 | □󠆯 | □󠆯 | □󠆯 | □󠆯 |
| 24. Upper limb complaints (e.g. trembling, cramps, lack of strength, sensitivity) | □󠆯 | □󠆯 | □󠆯 | □󠆯 | □󠆯 |
| 25. Memory /concentration disorders | □󠆯 | □󠆯 | □󠆯 | □󠆯 | □󠆯 |
