# Supplementary material for: Development and validation of TreatHSP-QoL: a patient-reported outcome measure for health-related quality of life in hereditary spastic paraplegia
Source: Orphanet J Rare Dis. 2024 Jan 2;19:2. doi: 10.1186/s13023-023-03012-w (PMC10763482; doi:10.1186/s13023-023-03012-w)
Supplement: Supplementary file 6 — Additional file 6: Score calculation for TreatHSP-QoL. [file 13023_2023_3012_MOESM6_ESM.docx]

**Additional file 6. Score calculation for TreatHSP-QoL**

All items of the questionnaire are five-level Likert items with values between zero and four. The questionnaire score, total or per subdomain, can be calculated by the mean value of all answered items times 25, i.e.,

$$Score= \frac{1}{K}\left( \sum_{k=1}^{K} X_{k} \right)\times25=\frac{1}{K}\left( X_{1}+X_{2}+\ldots+X_{K} \right)\times25$$

where $X_{k}$ represents the value for item $k$ with $K$ being the total number of answered items (or items that can be counted as answered, see explanation in the next paragraph). Thus, if the patient (or caregiver) answers all items with the best possible answer, the score (total of per subdomain) would be equal to $4\times25=100$. If the patient (or caregiver) answers all items with the worst possible answer, the score (total or per subdomain) would be equal to $0\times25=0$. The calculation of the mean implies that missing values (i.e., not answered questions) do not have a negative impact on the total score value. However, if less than half of the items in total (or per subdomain) are missing, the (sub-)score should not be calculated as the results would not be reliable.

The final questionnaires for patients and caregivers contain each 26 items. Due to applied filter questions, patients are distinguished between “working”, “non-working due to the disease”, and “non-working for another reason”. Depending on the occupational situation, one would be referred to a different item, so that a maximum of 25 out of 26 items could be answered by each participant. If crossing “non-working for another reason”, the patient (or caregiver) would skip the item regarding work. However, this skipped item still counts as the best possible answer, i.e., a value of four, as this implies they are not having any limitations due to HSP. Please note that the same approach was applied for items with filter questions in the questionnaires for the main validation step.

***Example score calculation***

A patient who is not working due to another reason than HSP can answer up to 24 items. Out of these 24 items, the example patient answered 19 items, so that five items were not answered. A value between zero and four can then be assigned to each of the 19 items, where zero represents the worst possible answer in terms of HRQoL and four the best possible answer. The sum of these 19 values equals in this example 56. Since the “non-working for another reason” counts as an item with the best possible answer, one has to add four points to this sum. As there are now twenty items that are counted for, the score can be calculated by

$\frac{1}{20}\times60\times25=75$.

Another example patient is a working patient, who has answered all of the 25 items with a sum of 63. For this patient, the score would equal the sum of 63, since

$\frac{1}{25}\times63\times25=63$.
