# Supplementary material for: Development and validation of TreatHSP-QoL: a patient-reported outcome measure for health-related quality of life in hereditary spastic paraplegia
Source: Orphanet J Rare Dis. 2024 Jan 2;19:2. doi: 10.1186/s13023-023-03012-w (PMC10763482; doi:10.1186/s13023-023-03012-w)
Supplement: Supplementary file 7 — Additional file 7: Interview guide, translated here from German to English for illustrative purposes. [file 13023_2023_3012_MOESM7_ESM.docx]

**Additional file 7.** Interview guide**,** translated here from German to English for illustrative purposes.

**1. Symptoms and associated limitations**

1.1. gait disturbance/leg weakness/leg spasticity

1.2. pain

1.3. bladder disturbances

1.4. other motor disorders

1.5. sensory disorders

1.6. cognitive disorders

1.7. epilepsy

**2. General health/quality of life**

2.1. vitality (energy/fatigue)

2.2. social life/environment

2.2.1 stigmatization

2.2.2. partnership

2.2.3. social participation

2.2.4. support from family and friends

2.3. professional life / occupational situation

2.4. leisure activities

2.5. psychological well-being

2.5.1 stress

2.5.2. emotional experiences

2.5.3. concerns about the disease / future

2.6. independence

2.6.1. mobility

2.6.2. household

**3. Satisfaction with treatment**

3.1. treatment options

3.2. medical care/health professional as a contact person

3.3. individual successes/failures

**4. Free associations**

4.1. future

4.2. family

4.3. quality of life

4.4. legs

4.5. doctor

4.6. aids

4.7. money

4.8. support

4.9. anxiety

4.10. depression

4.11. hope

4.12. dream

**5. Debriefing**

Thoughts on the interview in general

Comprehensibility

Relevance and usefulness

Appropriateness

Forgotten / not mentioned topics
